# Beyond Classic Anastomoses Training Models: Overview of Aneurysm Creation in Rodent Vessel Model

**DOI:** 10.3389/fsurg.2022.884675

**Published:** 2022-04-18

**Authors:** Pablo García Feijoo, Fernando Carceller, Alberto Isla Guerrero, Miguel Sáez-Alegre, Maria Luisa Gandía González

**Affiliations:** Department of Neurosurgery, La Paz University Hospital, Madrid, Spain

**Keywords:** microsurgery, anastomoses, model, neurosurgery, vascular, training, rodent

## Abstract

Nowadays, due to the decline in the number of microsurgical clippings for cerebral aneurysms and revascularization procedures, young neurosurgeons have fewer opportunities to participate and train on this type of surgery. Vascular neurosurgery is a demanding subspecialty that requires skills that can only be acquired with technical experience. This background pushes the new generations to be ready for such challenging cases by training hard on different available models, such as synthetic tubes, chicken wings, or placenta vessels. Although many training models for vascular neurosurgery have been described worldwide, one of the best is the rodent vessels model. It offers pulsation, coagulation, and real blood flow conditions in a physiologic atmosphere that mimics perfectly the intracranial human vessels environment, especially in terms of size. However, the current differences in governmental different regulations about the use of living animals in medical experimentation and the social awareness, as well as the lack of financial support, cause more difficulties for neurosurgeons to start with that kind of training. In this review, we describe the tools and techniques as basic steps for vascular microsurgery training by using rodent models, that provide an accurate copy of brain vessels environment under stable conditions. The initial three classical known microanastomoses for neurosurgeons are end-to-end, end-to-side, and side-to-side, but in literature, there have been described other more complex exercises for training and investigation, such as aneurysm models. Although there is still little data available, we aim to summarize and discuss aneurysm's training models and reviewed the current literature on the subject and its applications, including a detailed description of the techniques.

## Introduction

Since the endovascular era breakout, a global trend of decreased number of clipping and by-pass procedures can be appreciated worldwide (1, 2). On the one hand, there is a higher proportion of giant and complex aneurysm cases that are not suitable for training (3). On the other hand, many of the neurosurgeons who belong to the new generations have neither participate nor witnessed a complex clipping or a by-pass procedure, although these techniques may be necessary sometime during their career. Furthermore, due to the decline of microsurgical-related procedures, and under these hard conditions, the development of models that mimic intracranial human vessels (4) becomes the best way to be prepared. The time spent on that kind of training could be perceived by the trainee as a waste of time, taking into account the current varied range of techniques which they must master. Considering this situation, is it is a question of whether it is then worth investing time in neurovascular procedures training?

Surgical learning was long based on experimental models, which are fundamental for acquiring the necessary skills to perform safe surgical procedures in the real world. Probably, training on vascular models is one of the most complete trainings for neurosurgeons. These models not only ameliorate vascular skills, but they help to improve microsurgical dissection skills, tiny vessels manipulation micro-technique, and fine suturing (5, 6). Moreover, microsurgical training differs from other macroscopic models on the use of proper instruments, the average size of the treated structures, and the indirect field of view, highlighting the value of hand-eye coordination (or better said: “hand-brain”), a standard of microsurgery.

On this basis, the previous question has an obvious answer, yes. This training constitutes an extra instruction to carry on safely both the common and unusual complex procedures in which the use of microsurgical technique may be necessary.

Progressive learning in microsurgery connotes practicing on different types of models until mastering the technique that allows the trainee to comfortably reproduce similar procedures *in vivo*. Classically, in the beginning, *ex vivo* models, such as plastic tubes, human placenta (7), pig coronaries, or chicken wings have been recommended (8), and recently, thanks to virtual reality some novel simulated models are being explored, promising to bring shortly big advances in the field (9–12).

Despite the latest progress, one of the most accepted and complete models to train on vascular procedures are still the living models (4–6, 13), which offer a real blood flow environment accompanied by pressure, pulsatility, hemostasis, and an angioarchitecture that resemble the human intracranial conditions, being nowadays considered quite superior to the other available non-living models.

In that sense, the living rodent vessels is an optimal model that meets several requirements for high-quality training, and that has been historically widely used by neurosurgeons (4, 5, 14).

The recent recommendations of the European Society for Surgical Research (ESSR) and the International Society for Experimental Microsurgery (ISEM) (15) concerning the animal use for microsurgical training bet for the reduction and the rational use of the number of animals, however, there is a lack of international standardization in the matter, being this issue one of the main worries. To overcome these concerns, some basic experimental microsurgery principles have been developed: 3Rs [Replacement, Reduction, and Refinement, proposed by Russell and Burch in 1959 (16)], 3Cs [Curriculum, Competence, and Clinical Performance, proposed by Kobayashi and Lefor (17)], and GLP (Good Laboratory Practice).

At present, the aforementioned principles are essential for the justified and rational use of animals, more than ever before, taking into account the moral and legal considerations surrounding animal welfare in experimentation. The microsurgery trainees must be enrolled in a training program in which they can acquire the knowledge, aptitudes, and skills for the safe practice of animal experimentation.

The next question to be done is regarding the goal of using these models: whether we want to use them purely for training, or if we can also use them for basic investigation in aneurysm subject. In this sense there is a wide range of techniques described throughout history, most of them explained on macroscopic models or big animals (6, 18). Fortunately, the main basic principles can be exported and adapted to other microsurgical models. Our aim is not only to summarize the different aneurysm models, but also to perform a critical review and compare the different techniques, focusing on rodent vessels model, in which we have a great experience to share from our institution.

## Experimental *in vivo* Aneurysm Development

Many animals' intracranial aneurysm (IA) models have been tested since the first animal experiments were carried on in the early 60s (19). Throughout history, there have been attempts for improving the available IA models by using different strategies, technologies, and animal species. Currently, there are two different classes for experimental development, considering the mechanism of production (20):

- Aneurysm induction models: the aneurysm is obtained by generating the different risks factors that favor its endogenous formation.
- Aneurysm creation models: the aneurysm is crafted by direct microsurgical anastomoses.

Aneurysm models can also be classified according to their anatomical location (18):

- Intracranial models: more suitable to evaluate IA pathobiology.
- Extracranial models: ideal to test endovascular novel therapies and to train microsurgical skills.

**Figure 1:**
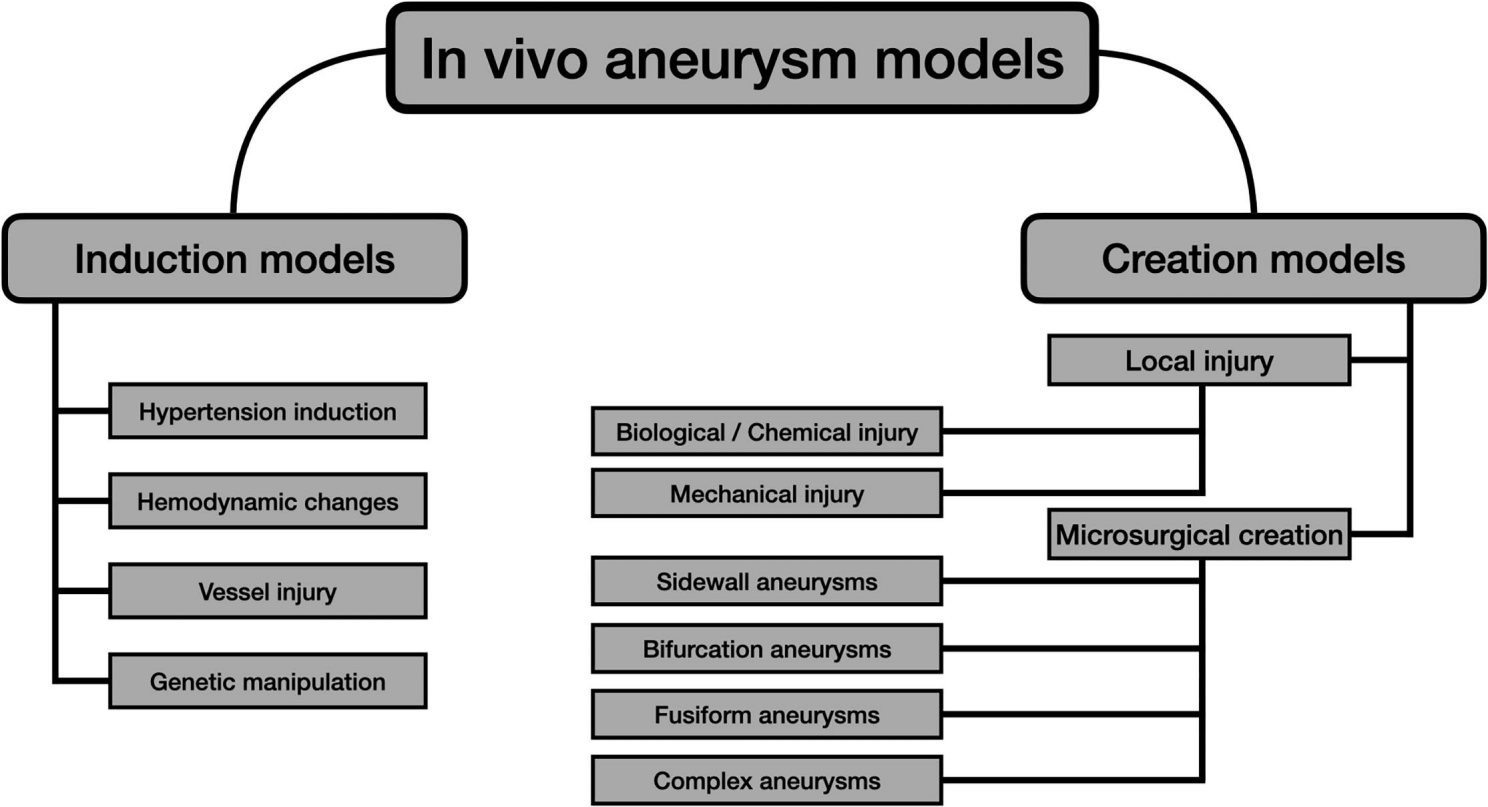
Diagram showing the most common experimental *in vivo* models for IAs.

Figure 1 summarizes the main strategies for the development of experimental *in vivo* aneurysms, considering the aforementioned issues.

The sections below provide a wide summary of both conceptions, also offering a critical review of their technical aspects and limitations, focusing on the microsurgical performances' tenets.

### Aneurysm Induction Models

Due to the several implications of IA rupture, there is a growing interest in their pathogenesis, many times focused on the identification of potential therapeutic targets or an early diagnosis that could avoid the potential devastating consequences following a subarachnoid hemorrhage (SAH) (10, 21).

A historical preference for the use of rodents can be observed, and this trend answers to some reasons such as the similarities between mice and humans in terms of aneurysm location and pathology, including degradation of the internal elastic lamina and thinning of the tunica media. The lower costs and the quick production rate of the animal cohort also make them ideal for these purposes (18, 22).

Despite the high degree of histological similarities, the incidence of natural SAH in mammals other than man is ultra-low (22), being only described in chimpanzees (*Pan Troglodytes*). Consequently, a true aneurysmatic SAH is also a rare entity to find in controlled murine cohorts, where even the presence of induced IAs makes this rupture difficult to predict or synchronize (23). Hence the preference of the direct blood injection into the subarachnoid space or the endovascular perforation of a cerebral vessel as a model for SAH.

On these grounds, nowadays, the precise mechanisms underlying IAintracranial aneurysm formation and rupture remain unclear, although most of the induction models are based on three main principles which can also be found in humans acting as risk factors: hypertension, hemodynamic changes, and vessel injury. Moreover, estrogen deficiency and genetic manipulation are increasing impact on all recent reviews (20, 24). The combination of at least 2 or 3 of these methods is often described in the literature to improve the success rate, however, there is no international consensus about which is the best association.

One of the earliest experiences to induce IAs was reported in 1961 by White JC, by sodium chloride hypertonic (28%) solution injections in the internal carotid artery wall of mongrel canines (19). New agents are lately being explored, oriented to the weakness or degradation of the intracranial arteries connective tissue layers, especially the internal elastic lamina.

One example is elastase, which can be administered stereotactically into the basal cisterns. This enzyme is often combined with an angiotensine-II subcutaneous infusion through an implanted osmotic pump to increase hypertension, augmenting the anterior circulation IA incidence to 70% of the specimens (25).

In most of the surgical hypertension-induced based models, branches of the renal arteries are surgical ligated (26, 27), the rat is nephrectomized or its kidney is wrapped (20). According to this strategy, high-salt diet has been widely used. Deoxycorticosterone acetate-salt administration may have a dose-dependent effect, influencing both aneurysm formation and rupture (23, 28). Despite being one of the favorite methods, by itself, the hypertension induction model is not enough to get an acceptable IA incidence rate (20).

In 1978, the Hashimoto model for experimentally induced IA was described (29). This model aimed for the loading hemodynamic stress to damage arterial walls; a conception that persists to this day with slight modifications as one of the most important milestones in the field. Originally, Hashimoto (29, 30) fed the rats with beta-aminopropionitrile to weak the cerebral arterial wall, and through a unilateral common carotid artery (CCA) ligation, in addition to deoxycorticosterone infusion and salt-diet, the saccular IA incidence rate was increased, showing validity as a model for saccular cerebral aneurysms in humans afterward (31, 32), and exporting the model to other species such as monkeys (33).

**Figure 2:**
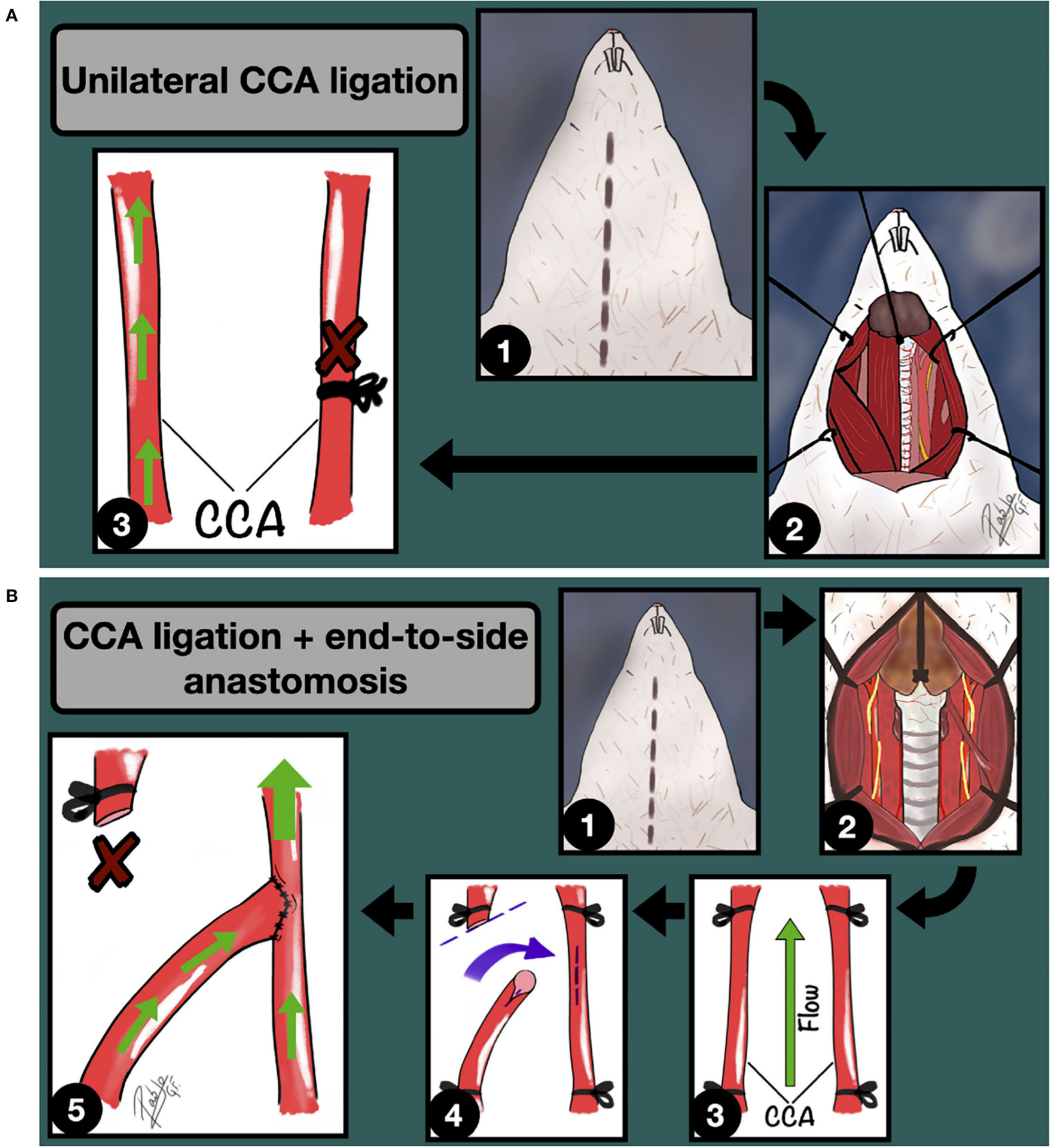
**(A)** Unilateral CCA ligation model for IA induction through hemodynamic stress increase. 1: Midline cervical incision; 2: Cervical vessels approach and unilateral CCA exposure; 3: Unilateral CCA ligation. The green arrows indicate the blood flow direction. **(B)** Unilateral CCA ligation + Contralateral End-to-side anastomosis model for IA induction through hemodynamic stress increase. 1: Midline cervical incision; 2: Cervical vessels approach, focusing on the left CCA; 3: CCA clamping; 4: Longitudinal arteriotomy of the parent artery and fish-mouth arteriotomy of the donor vessel; 5: End-to-side anastomosis is completed. The green arrows indicate the blood flow direction.

Then, in the late 80s, Roda and Alvarez (26, 27) detailed better the influence of the hemodynamic stress on the IA incidence rate in rats, succeeding almost three times more in those animals which developed a higher carotid artery flow, thanks to the combination of unilateral CCA ligation and an end-to-side anastomosis between carotids, in comparison to those which underwent only unilateral CCA ligation (Figure 2). Further, they demonstrated how this combination increased the blood flow through the parent carotid artery up to 89% in 5 months, inducing intracranial vessels damage (34). Other modifications from the original model describe the ligation of the external carotid artery and/or pterygopalatine artery (which, in rats, arises more proximal than in humans) to rise the hemodynamic stress through the internal carotid artery (33, 35).

Recently, genetic modification is a promising emerging strategy to improve the IA incidence rate, often based on some human conectivopathies features and hypertensive mechanisms (20). Transgenic mice with altered specific proteins which are supposed to play an important role in IA formation, such as eNOS and SOX17, can be purchased (36). However, as in humans, genetic predisposition by itself is not enough to guarantee IAs obtention, so the knock-out individuals must be further subjected to a combination of some of the previously detailed methods (20).

The size and characteristics of the obtained aneurysms differentiated them from the real ones found in humans, being quite common to observe smaller aneurysms with shallow/wide necks and aneurysmal bulges with a less rounded shape (20).

In spite of all this, at present, the most common technique employed to induce IAs in rodents is still the surgical ligation of CCA and/or renal artery (uni or bilateral) with concomitant induction of hypertension (37).

The principal described induction models for IAs are neither efficient nor useful for training microsurgical skills due to their average volumes and long induction time. Notwithstanding the foregoing shortcomings, some parts of these models require microsurgical skills (end-to-side anastomosis between CCAs or the renal arteries ligation) that can be valuable for training purposes.

### Aneurysm Creation Models

After the provided brief summary of the induction models, the pure microsurgical models, which are the main objective of the present review, will be detailed in the sections below. The diagram shown in Figure 1 divides the creation models into two groups: local injury and microsurgical models.

Unlike the induction models, the microsurgical creation models are preferentially located on extracranial vessels (36) due to their bigger diameter and easier approach. This fact becomes even more relevant considering that the rodent extracranial vessels caliber is very similar to many segments of human intracranial vessels from both anterior and posterior circulation (14).

A recent systematic review (18) on preclinical extracranial aneurysm models, found more than 68 described models/techniques in five different species and confirmed that the most widely used animals for the microsurgical aneurysm creation are mice, rats, dogs, swine, and rabbits, being these models less frequently described on sheep and monkey species.

**Figure 3:**
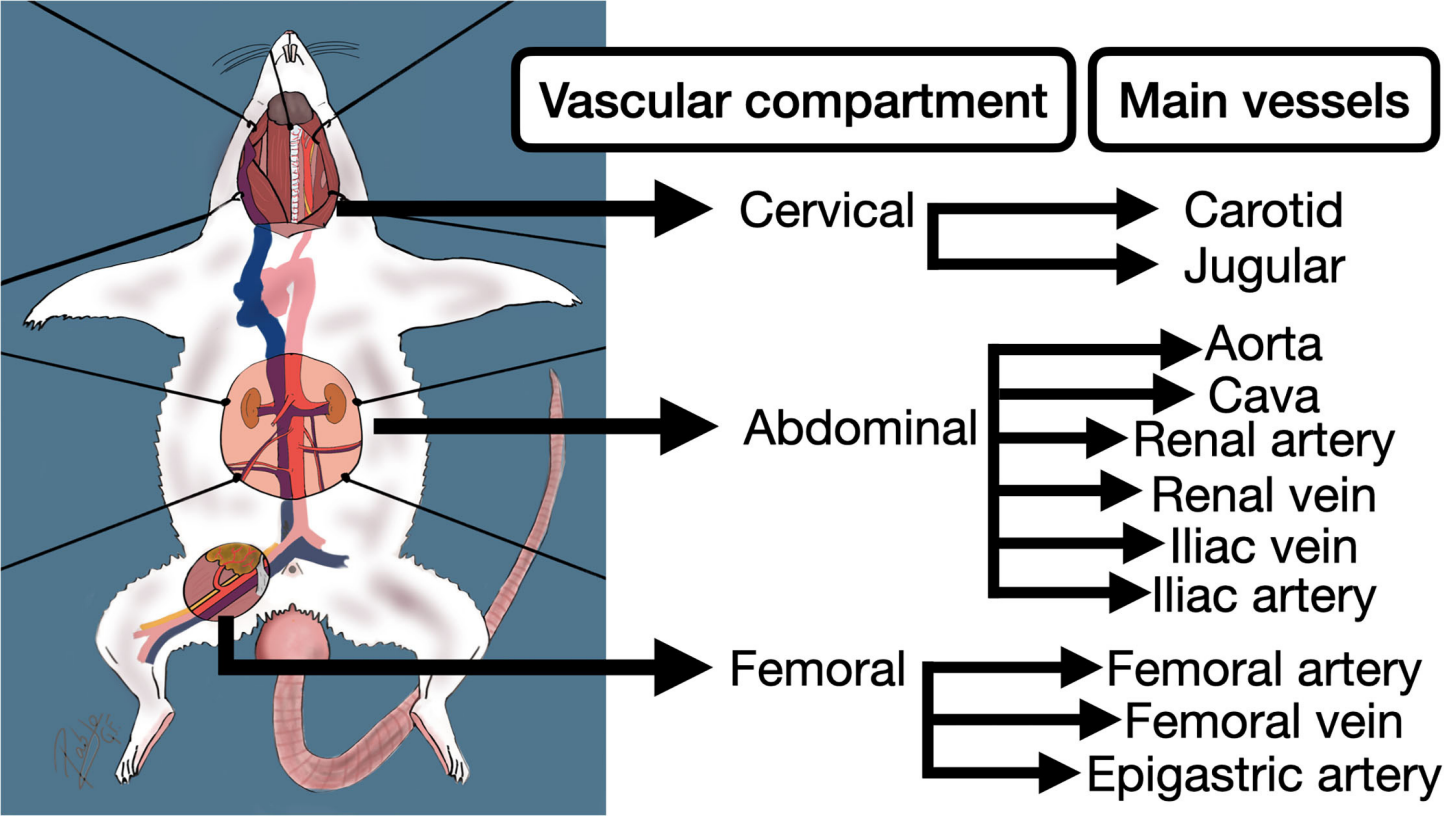
Principal vascular compartments and vessels of the rodent for the practice of anastomoses in extracranial models for aneurysms.

It is also important to understand the rodent's internal anatomy and vessel approaches, that many times differs from human (5). The vascular anatomy of the rat, in terms of vascular training, can be divided into three main compartments: Cervical, abdominal, and femoral (Figure 3). Each compartment exhibits different characteristics related to its anatomical location. For instance, the cervical compartment is more related to the trachea and head structures, in the abdominal compartment the main vessels are surrounded by many organs and fatty tissue, and the femoral compartment offers two tiny parallel vessels accompanied by the femoral nerve lying on a muscle background.

There are three basic concepts that the surgeon needs to bear in mind before starting the exercises: the size of the vessels, the nearby vessels, and the local anatomy of the vessel. The nearby vessels matter when a side-to-side or end-to-side anastomosis is needed (a large donor artery may be helpful for this exercises). Some special configurations of the local anatomy of the vessels are also useful when an anastomosis on a bifurcation or a terminal branch aneurysm are being planned.

There are four types of human IAs attending to their shape and walls: saccular, fusiform, dissecting, and mycotic. However, the most common type found in humans by far, which represents up to 90% in all reviews, is the saccular type (21, 38). For this reason, they are also the most reproduced worldwide as a model for training. Normal saccular IAs growing implies a degradation of the internal elastic lamina and thinning of tunica media, resulting in a sac formation that arises on the arterial wall, covered only by the tunica intima and adventitia (21). Several different techniques and constructions for mimicking the most frequent type of aneurysm have been described, however, it is still not well-established which is the gold standard.

Marbacher et al. (18) described and classified the preclinical models for IAs according to the main anastomosis combination or vessel occlusion employed and its similitude to human IAs into the following types: sidewall aneurysms, stump aneurysms, terminal aneurysms, bifurcation, and other complex aneurysms. The original descriptions in the literature include some endovascular-based models and were made on different animal species, therefore, not all these performances can be adapted to the present model. For academic reasons, and aiming to explore and describe the rodent models for IAs, attending to pure microvascular training objectives, we find it simpler to classify them into four major classes as follow:

1. Sidewall aneurysms
2. Bifurcation aneurysms
3. Fusiform aneurysms
4. Other complex aneurysms.

This proposed classification emphasizes the location of the aneurysm relative to the parent artery and the named subclasses provide a quick conceptual explanation about the technique.

On the other hand, some reported creation models for IAs are not exclusively microsurgery-based, although they suggest some surgical steps different from anastomosis to induce a local injury. This is the case of the fusiform aneurysms created by a CCA isolation followed by local application of elastase (4, 39).

All the exercises must be done under the legal qualification, taking into account animal welfare. In that sense, all the procedures included in the present review, to illustrate the following techniques, were performed at idiPAZ microsurgery laboratory, at our institution, by accredited trainees, under the European directive EU 63/2010 and were approved by an Animal Ethics Committee under PROEX 160/17 according to RD 53/2013 (Spain).

#### Sidewall Aneurysms

Sidewall aneurysms are considered the most feasible model to be performed, being the first recommended exercise for vascular training within aneurysms. The concept comprises the obtention of a rounded or tubular aneurysm arising from the lateral wall of the parent vessel, which provides a blood inflow into this outpouching portion. The resulting constructions can be easily identified as aneurysms, resembling those that can be found in human intracranial vessels. However, the surrounding tissue differences in comparison to the real IAs, which are located into the deep subarachnoid space (between brain parenchyma), is one of the obvious limitations of all the extracranial models.

The four designed strategies listed below are considered easy to reproduce, considering that only one functional vessel is involved in the performance and that the graft, when needed, is obtained from another expendable vessel, which allows the practitioner to keep undamaged the rest of the circulatory apparatus. Furthermore, the sidewall class can be practiced on the three mentioned vascular compartments of the rat.

##### End-to-Side Graft Placement

**Figure 4:**
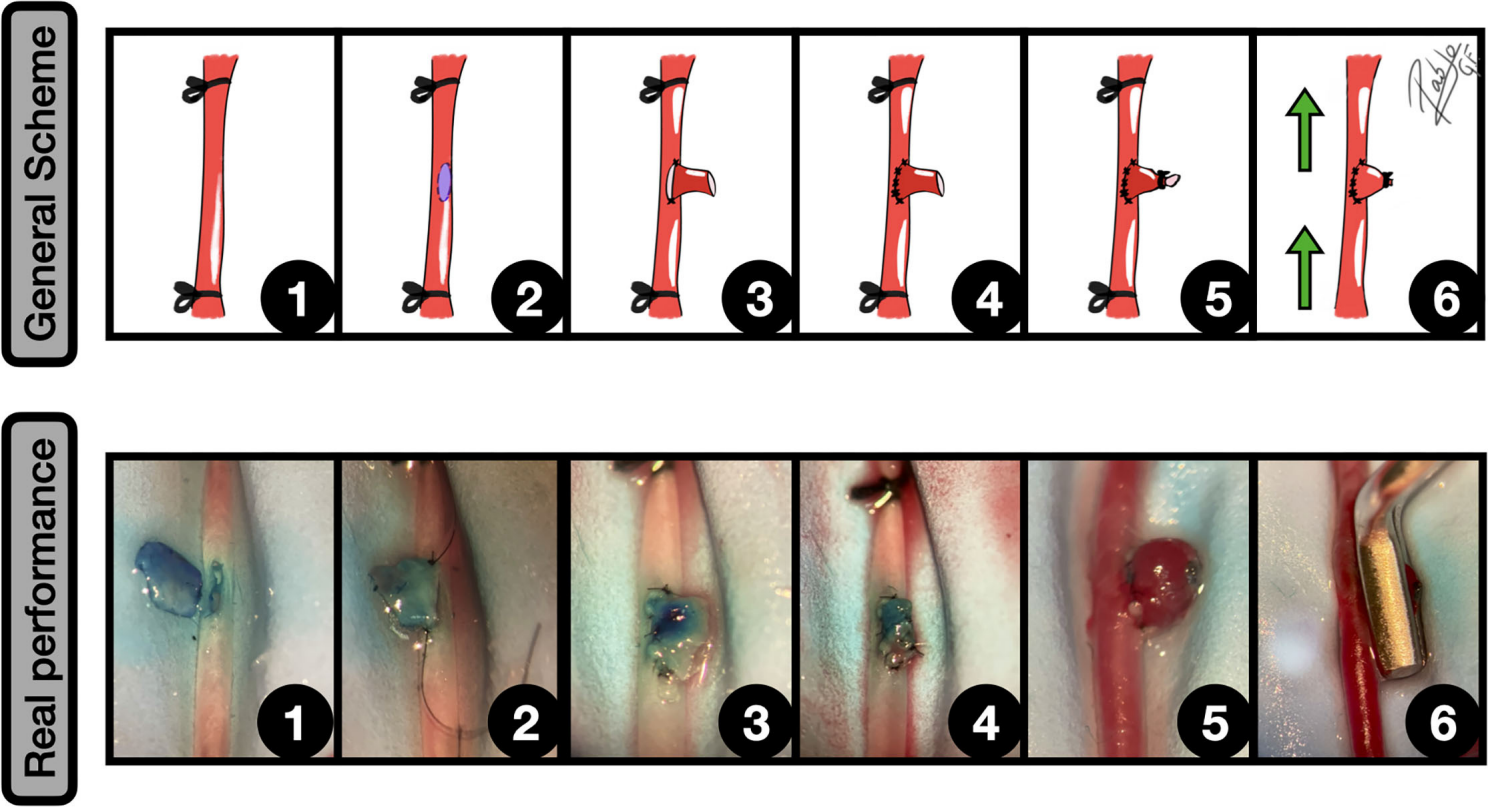
End-to-side graft placement. General scheme: **1:** CCA exposure and proximal and distal clamping; **2:** Lateral wall excisional arteriotomy of the CCA; **3:** The vein graft is placed against the arteriotomy edges, and the two first anchoring knots are made; **4:** End-to-side anastomosis between the graft and CCA is completed; **5:** The free lumen of the graft is occluded; **6:** After clamping releasing an inflow can be observed into the aneurysm. The green arrows indicate the blood flow direction. Real performance: **1:** Excisional arteriotomy on the *antero-lateral* wall of the CCA. A blue-dried venous graft harvested from a jugular vein is on the left; **2:** The graft is fixed to the arteriotomy by a couple of anchoring knots; **3:** The arteriotomy is fully anastomosed to the graft in an end-to-side fashion; **4:** The free lumen of the graft is occluded by an uninterrupted suture; **5:** After releasing the clamps an inflow is observed into the venous graft, simulating a sidewall aneurysm; **6:** The aneurysm is clipped, preserving the parent vessel circulation.

Most of these vascular performances imply an end-to-side anastomosis between a parent artery and a vascular graft, venous or arterial, that can even be artificial. The obtained performance is a rounded vascular formation, that arises from the lateral wall. Depending on the graft material, different variations in terms of final size can be achieved. In our experience, the venous grafts can undergo significant growth that starts some minutes after the declamping and may get a huge size after some days. For this reason, arterial grafts are preferred when standardization is needed (18, 40), since the aneurysm final size is better kept and controlled. According to the Helsinki model (40), this modality is most of the times described for the abdominal aorta or the carotid (6), following these common steps (Figure 4):

1. Aorta dissection and subsequent distal and proximal clamping
2. Excisional arteriotomy on the lateral wall, inner arterial flushing with saline, and edges dying
3. Preparation of the graft and placement of the first two anchoring knots of an end-to-side anastomosis
4. End-to-side anastomosis is completed by the rest of the knots
5. The free lumen of the graft is occluded
6. The normal circulation is restored by clamping releasing.

The previous availability of the graft shortens the surgical time, however, to attempt a most challenging training it is recommended to harvest the graft microsurgically from nearby structures, such as renal vessels or cava (in the abdomen) and the CCA and jugular vein (in the cervical compartment) (6, 40).

The infrarenal segment of the aorta is preferred by many authors because of the fewer number of branches, however, there is a disturbing bilateral major branch called the iliolumbar artery, easy to confuse with the renal arteries and that must be often ligated (5, 40).

##### Side-to-Side AVF

**Figure 5:**
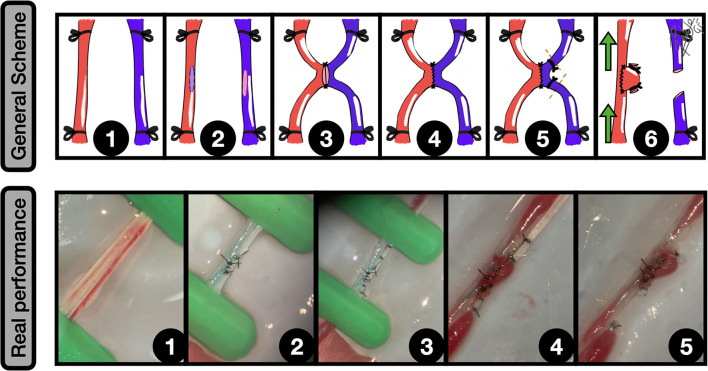
Side-to-side arteriovenous fistula (AVF). General scheme: **1:** Femoral artery and vein are widely exposed and proximal and distal clamped; **2:** Lateral wall longitudinal arteriotomies of both vessels are performed; **3:** The two first anchoring knots are made, bringing both vessels together; **4:** Side-to-side anastomosis between artery and vein is completed; **5:** A double ligature of the vein, near to the anastomotic zone is made, and a subsequent section is performed to create the dome of the aneurysm from this vessel; **6:** After clamping releasing an inflow can be observed into the aneurysm. The green arrows indicate the blood flow direction. Real performance: **1:** Femoral artery and vein exposure and clamping; **2:** Side-to-side anastomosis; **3:** Distal and proximal femoral vein ligatures, to ensure a proper segment length for the aneurysm; **4:** The clamps are released and the isolated venous segment receives an inflow from the femoral artery, which remains between the ligature limits; **5:** The distal and proximal ligated segments of the femoral vein are sectioned, obtaining a sidewall aneurysm that arises from the femoral artery. Mild vasospasm can be noticed.

In principle, this model is more challenging than the previous, since the proposed anastomosis is a side-to-side which involves two parent vessels. They cannot be as comfortably handled as a free graft and are often from different natures (artery and vein is a common combination). After the anastomosis is performed, the vessel that will recreate the aneurysm must be sacrificed by distal and proximal ligation (6, 18). Planning is essential to achieve the best results because the nearby vessels' location must be prior recognized for further considerations about their transposition. Besides the vessels' proximity in the femoral compartment facilitating the exercise, the mistakes in this area do not usually result in fatal hemorrhage (41). The recommendations for the cervical compartment suggest a jugular vein transposition, although, despite the name, this model can be executed by a side-to-side anastomosis between two arteries, such as CCAs. The basic steps are (5, 6, 18) (Figure 5):

1. Parallel vessels dissection and clamping
2. Incisional arteriotomy on the anterolateral wall of both vessels, inner arterial flushing with saline, and edges dying
3. Anchoring knots must be placed on the arteriotomy cranial and caudal edges
4. Side-to-side anastomosis by suturing the two resulting walls in a stepwise manner
5. Delayed proximal and distal ligation and section of the recipient artery
6. The sidewall aneurysm is obtained when the blood flow is restored through the parent vessel.

##### Terminal Branch Occlusion Model

**Figure 6:**
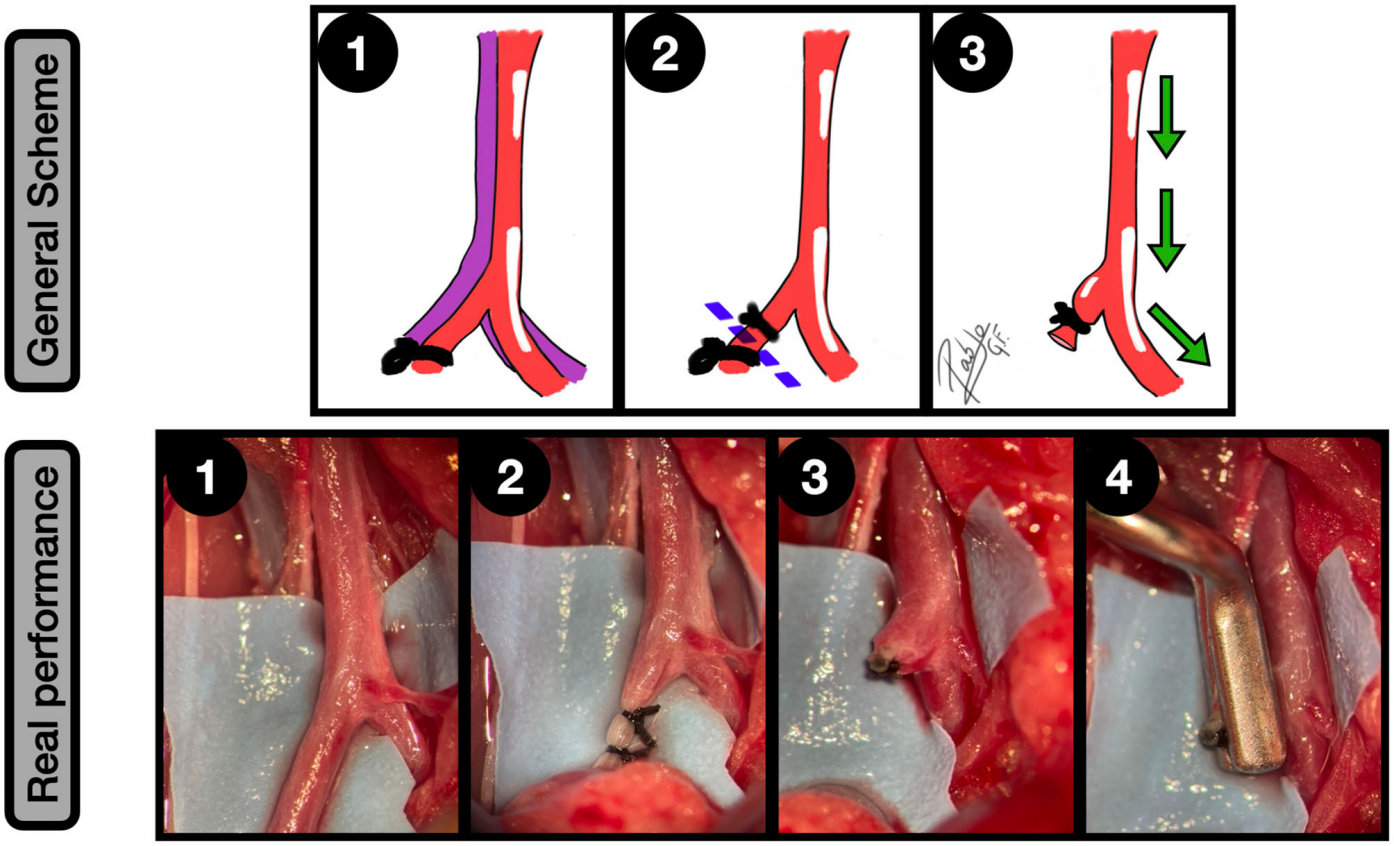
Terminal branch occlusion model. General scheme: **1:** AIB is dissected from the cava and one of the common iliac arteries is ligated; **2:** The common iliac artery is sectioned between the two ligatures, closed to the bifurcation; **3:** The remaining branch of the common iliac artery simulates a sidewall aneurysm while the aorta and contralateral common iliac artery become the parent vessel. The green arrows indicate the blood flow direction. Real performance: AIB and right common iliac dissection and exposure; **2:** Left common iliac artery ligation in 2 points, with 6/0 silk. The proximal ligature must be placed close to the aortoiliac bifurcation AIB as is shown; **3:** Common iliac artery section. A sidewall aneurism is obtained, acting the aorta and the contralateral common iliac artery as a parent vessel; **4:** The aneurysm is clipped.

The terminal branch model was originally described to investigate and test endovascular techniques (42), being the most feasible and simple to reproduce in the laboratory. It consists of a surgical arterial occlusion, which is not technically demanding for the trainee. The obtained aneurysm can be a sidewall or a stump aneurysm depending on the parent vessel location and flow direction, and thereby, it constitutes a good model for terminal aneurysms too. The best locations, when a sidewall aneurysm is planned by this method, are closed to natural bifurcations, where the occlusion of one branch, some millimeters far from the divergence, allows to keep the other one patent, simulating a sidewall aneurysm. The few steps needed are listed below (Figure 6):

1. Natural bifurcation exposure and dissection of the sacrificed vessel
2. Ligation of one of the vessels near to bifurcation
3. After a distal section of the vessel, the aneurysm is obtained.

##### No-Neck Sidewall Aneurysms Model

**Figure 7:**
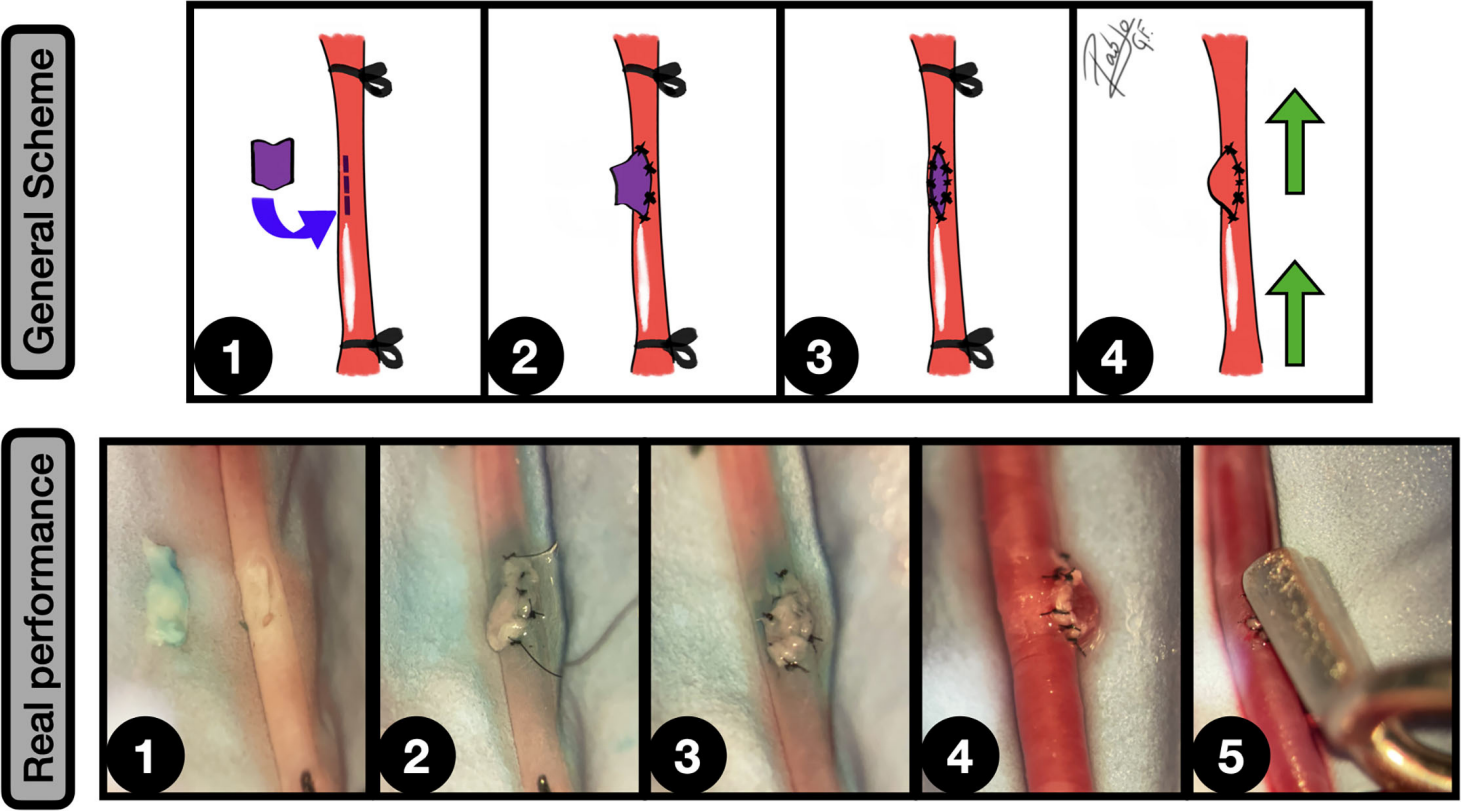
No neck sidewall aneurysms model. General scheme: **1:** CCA is exposed and proximal and distal ligated. A longitudinal arteriotomy in the lateral wall is performed and a plain venous graft from a jugular vein is harvested; **2:** The graft is fixed to the arteriotomy edges by a couple of key anchoring knots; **3:** The arteriotomy is totally covered by the venous graft in an end-to-side fashion; **4:** After clamping releasing an inflow can be observed into the aneurysm. The green arrows indicate the blood flow direction. Real performance: **1:** A longitudinal arteriotomy on the CCA anterior wall is performed. A plain venous graft, obtained from a jugular vein is shown on the left; **2:** The graft is placed on the arteriotomy and fixed by some anchoring knots; **3:** The graft is fully anastomosed on the arteriotomy, using interrupted stitches; **4:** Clamps are released, appreciating a blood inflow into the graft, which simulates a no-neck sidewall aneurysm; **5:** The aneurysm is clipped, preserving the parent vessel circulation.

This model is a variation from the end-to-side model that was related before. It is a simple design where a plain vein graft is sutured against a longitudinal incision in the arterial wall, obtaining a lateral wall aneurysm with no neck (43). It differs from the previously described models in the characteristics of the dome, which offers a more realistic, natural, and smooth surface since it does not need any suture or occlusion for its creation, as it is shown in Figure 7 following the next steps:

1. A plain vein graft is harvested from the jugular vein and a longitudinal arteriotomy in the CCA lateral wall is made
2. The vein graft is fixed to the arteriotomy by a couple of anchoring knots
3. The arteriotomy edges are totally sutured to the vein graft in an end-to-side fashion
4. The blood flow is restored, obtaining a saccular formation with no neck arising from the lateral wall.

#### Bifurcation Aneurysms

The second type of aneurysms existing in previously published literature refers to those which arise from an arterial bifurcation, which in real-world are often found in the middle cerebral artery (MCA), anterior cerebral artery, anterior and posterior communicating arteries. They represent one of the most common MCA IAs, accounting for up to 85–93% of cases (44) and up to 100% of the anterior communicating artery aneurysms (45), making the difference with their sidewall counterparts, which are more frequently located in the internal carotid artery (75.6%). This configuration is an independent risk factor for rupture (45), being preferred the surgical clipping rather than endovascular treatment by most of the reported case series (44, 45). However, both treatments in such vascular anatomic confluence can be hazardous when proper training is not at first carry on (46). The bifurcation models of IAs provide not only a realistic associated vascular circuit, but an aneurysm neck arising directly from the arterial flow divergence. One of the advantages of this model lies in its favorable hemodynamic, preventing thrombosis better than sidewall aneurysms, a characteristic that may be useful when long-term further analyses are needed (18, 46).

Most of these exercises were described on a swine (47), canine (46), or a rabbit model (48, 49), especially on the bifurcation between CCA and external carotid artery. Unfortunately, in rodents, this is not a feasible location for our purposes, although the basic principles can be tailored (6). The recommended vascular compartments depend on the nature of the bifurcation, dividing the currently published models into two major subgroups: natural bifurcation and neobifurcation aneurysms.

##### Natural Bifurcation Aneurysms

Natural bifurcation aneurysms need the suitable anatomy that a main arterial ramification offers. The abdominal compartment (Figure 3) contains the aorto-iliac segment, a major division of the aorta located below the ilio-lumbar vessels and followed by size by the renal arteries (5). The right ilio-lumbar artery normally arises closer (9.7 mm of distance) to the aorto-iliac bifurcation (AIB), being the distal aorta average caliber lower than proximal (1.2 vs. 2.4 mm), but similar to common iliac arteries as it is described in Sprague-Dawley specimen (14). This landmark is a critical structure whose manipulations may compromise seriously the posterior limb's blood flow, unlike the femoral artery. The complete occlusion of this last vessel preserves the flow thanks to an extra-abdominal anastomotic circle between the internal and external iliac arteries (6, 50).

**Figure 8:**
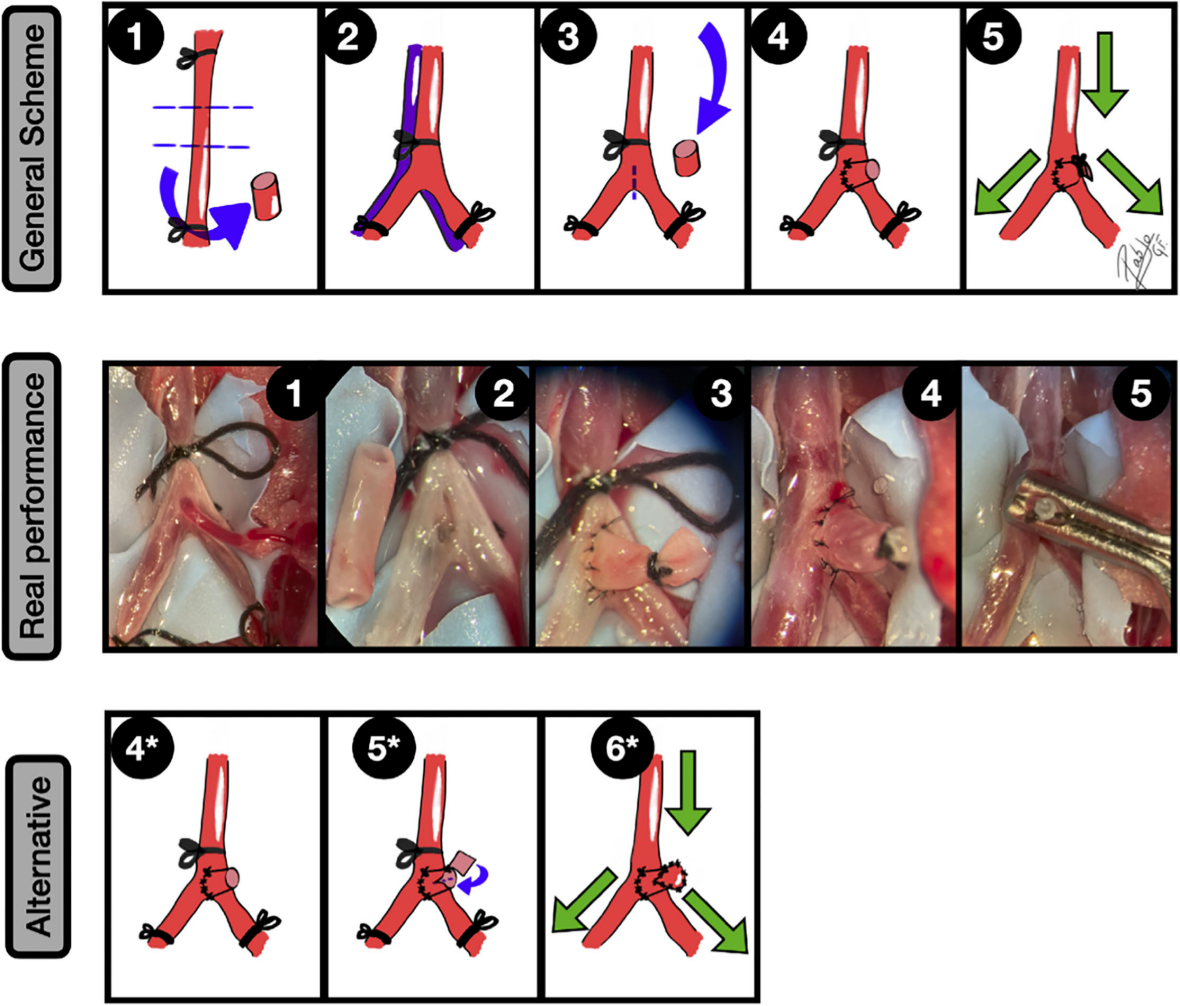
Natural bifurcation aneurysms. General scheme: **1:** A free CCA graft is harvested; **2:** The AIB is gently dissected from the cava vein and common iliac arteries, paying special attention to the posterior branches and subsequent ligation of the aorta and iliac veins is performed; **3:** A longitudinal arteriotomy is made in the anterior arterial wall, near to the bifurcation. After that, the CCA arterial graft is transferred to the abdominal compartment; **4:** An end-to-side anastomosis between the graft and the aorta is made; **5:** The free lumen of the graft is occluded and the clamps are released, leading to an aneurysm formation in the bifurcation. The green arrows indicate the blood flow direction. Real performance: **1:** Exposure of AIB over the blue contrast background. A posterior branch was ligated; The AIB is gently dissected from the cava vein and iliac arteries, paying special attention to the posterior branches, and subsequent ligation of the aorta and common iliac arteries is performed. In the picture, a third aortic caudal branch is observed; **2:** A longitudinal arteriotomy is made in the anterior arterial wall, near to the bifurcation, removing the tiny arterial branch. The CCA arterial graft is transferred to the abdominal compartment (left); **3:** An end-to-side anastomosis, by interrupted suture, between the graft and the aorta is made. A 6/0 silk ligature is performed to occlude the free lumen of the CCA graft; **4:** The clamps are released, leading to an aneurysm formation in the bifurcation; **5:** The aneurysm is further clipped. Alternative: 4*: End-to-side anastomosis between the CCA graft and the AIB; 5*: A fish-mouth arteriotomy is performed in the main CCA graft-free lumen, where a second plain arterial graft is fixed by an anchoring knot. 6*: The free lumen is fully covered by this plain graft and the clamps are released, obtaining thus a larger aneurysm. The green arrows indicate the blood flow direction.

The steps for its performance on rat AIB are (Figure 8):

1. Graft harvesting (CCA is highly recommended)
2. AIB dissection from the cava and common iliac arteries exposure
3. Longitudinal arteriotomy on the aorta anterior wall near the bifurcation
4. End-to-side anastomosis between the graft and the aorta
5. The free lumen of the graft is occluded by a ligature (alternatively by an additional graft) and the normal circulation is restored by the clamping releasing, obtaining the aneurysm.

Mücke (13) described this model by using 20 Wistar adult rats, highlighting a mean average volume similar to human IAs, the absence of thrombosis in the zone of central blood inflow into the aneurysm, and the control of the final dome dimensions thanks to a slight modification provided by a second graft to close the free lumen, instead of direct tighten by a ligature.

Care must be specially taken when the posterior wall of the AIB is dissected from the cava because the artery covers partially the view of the structures behind, where some posterior wall branches may be accidentally injured. To solve this challenge step, this posterior branch must be early identified by mild anterior and lateral transposition of the bifurcation. Secondly, a gentle dissection must be done before proceeding to its ligature.

Additionally, at this level more major arteries and veins than in other locations are present, making riskier the procedures in this area (51). This is the reason why this exercise is one of the most complete microsurgical trainings, combining dissection of an arteriovenous capital confluence, the transference of a free graft harvested from a distant vascular compartment, and the performance of an end-to-side anastomosis on an arterial confluence.

##### Neobifurcation Aneurysms

This term means the microsurgical performance of an aneurysm model placed on an artificially created vascular bifurcation. Considering that the bifurcation can be created by an end-to-side anastomosis between two nearby vessels, neobifurcation aneurysms can be either located on abdominal or cervical compartments. The steps are described for the CCAs (4, 6, 36, 46, 49).

**Figure 9:**
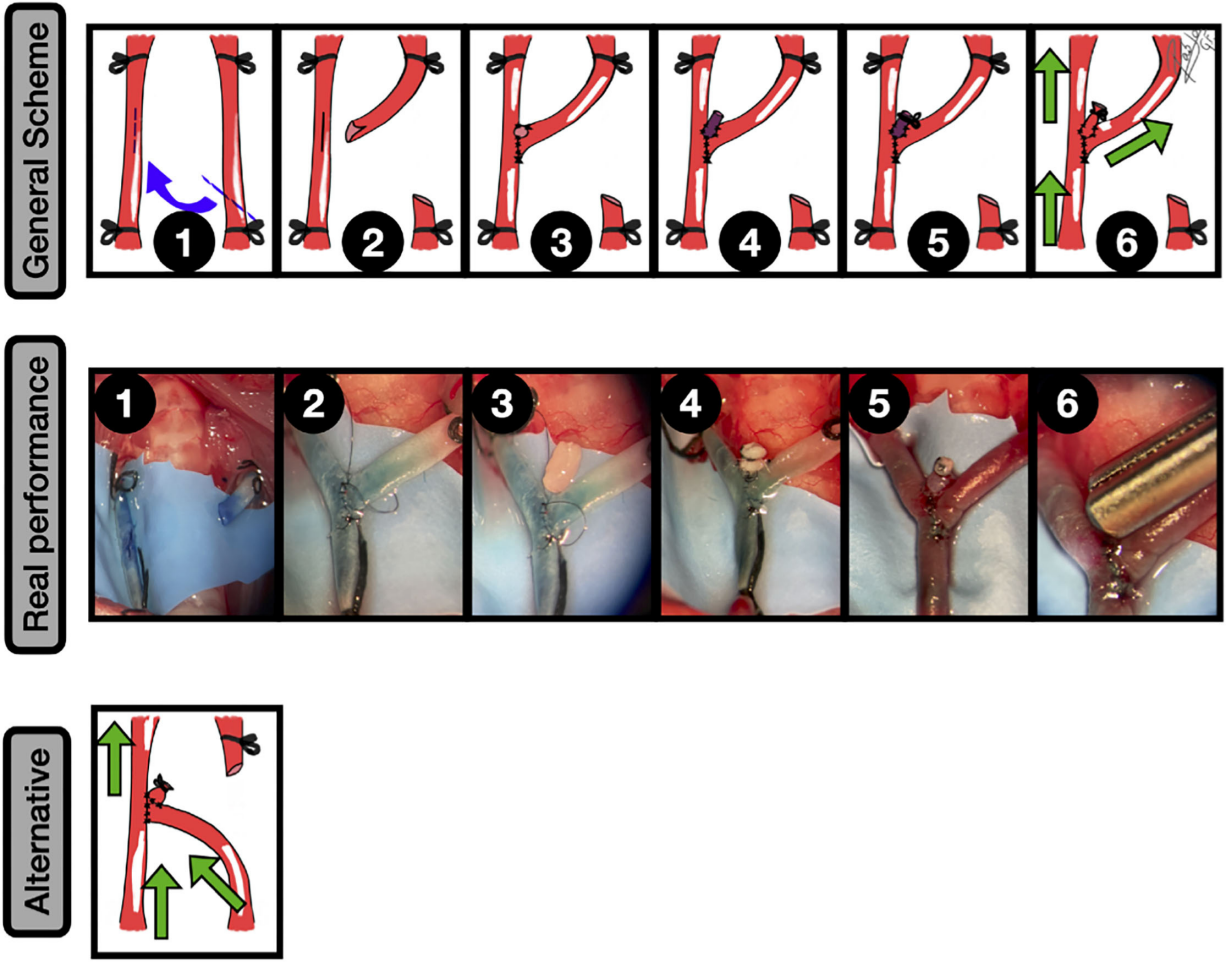
Neobifurcation aneurysms. General scheme: **1:** Bilateral CCAs exposure and proximal and distal ligation. A longitudinal arteriotomy in the anterolateral wall of the left CCA is done and an oblique arteriotomy facing the contralateral artery in the second CCA is performed; **2:** The picture shows the anastomosis construction and the fish-mouth arteriotomy in the recipient artery; **3:** An end-to-side anastomosis between both CCAs leaving a hole on the top is performed; **4:** A free venous graft from a jugular vein is placed on the top of the construction and anchored by a couple of knots; **5:** The anastomosis between the graft and the hole is completed and the free lumen of the graft occluded; **6:** The clamps are released, leading to an aneurysm formation in the bifurcation. The green arrows indicate the blood flow direction. Real performance: **1:** Exposure of both CCAs and subsequent arteriotomies in an end-to-side fashion, including a fish-mouth arteriotomy of the recipient artery (right). The vessels are dyed by methylene blue; **2:** The end-to-side anastomosis between both CCAs is performed, leaving a hole in the top that will be the neck of the aneurysm; **3:** A free venous graft from a jugular vein is harvested and transferred to the anastomotic area in the showed place; **4:** A second end-to-side anastomosis between the free graft and the hole is performed, and a 6/0 silk ligature is utilized to occlude the free lumen of the venous graft; **5:** The clamps are released, leading to an aneurysm formation in the bifurcation; **6:** The aneurysm is further clipped. Alternative: Confluence aneurysm by an end-to-side anastomosis between the CCAs + venous graft to recreate the aneurysm. The blood flow has a different direction from the aforementioned example. The green arrows indicate the blood flow direction.

In fact, this model conceptually consists of a modification of a simple end-to-side anastomosis as it is detailed (Figure 9):

1. Wide exposure of both CCA and graft harvesting from a jugular vein
2. Excisional arteriotomy on the anterolateral wall of the parent artery and oblique arteriotomy of the recipient artery
3. End-to-side anastomosis between both carotids, leaving an orifice in the corner which will provide blood a blood inflow into the future aneurysm
4. Suture of the previously obtained venous graft on the orifice
5. The free lumen of the graft is tightened by a ligature
6. The normal circulation is restored by clamping releasing.

It is important to ensure that a proper length of the involved arteries is taken with the aim of avoiding excessive tension in the anastomosis area. The jugular vein is probably the nearest and more suitable vessel to harvest in terms of size, and it must be mentioned that this venous pouch will lead to bigger aneurysm final average volumes in comparison to arterial grafts (36, 49, 52). This model is technically more challenging because the end-to-side anastomosis technique must be mastered, and the graft must be meticulously placed to avoid blood leakages. On the other hand, one of its main advantages is that the angle of bifurcation can be fashioned (47). There is even a model that mimics the human basilar tip aneurysms (6), where the bifurcation is artificially created by a side-to-side anastomosis between both CCAs and then the graft is sutured to an orifice on the top of the construction to simulate the dome.

In this category, another type of aneurysms must be named, due to the similarities in terms of the technical sequence: the confluence aneurysms. This model creates an aneurysm by a venous pouch placed on an end-to-side anastomosis, however, the graft must be located on a confluent blood flow construction instead of a divergence flow. For the CCAs, the conceptual change resides in the arteriotomy and the artery selection, where now the recipient artery becomes a donor (Figure 9). This means that the final construction offers two inflow vessels, remaining only one outflow vessel distal to the aneurysm. In general, high blood flow through the anastomosis is a positive factor for its patency (53). The confluence model mimics, for instance, the real-world hemodynamic conditions presented in human vertebrobasilar junction, where complex cases for both endovascular and clipping techniques are a challenge (46).

#### Fusiform Aneurysms

**Figure 10:**
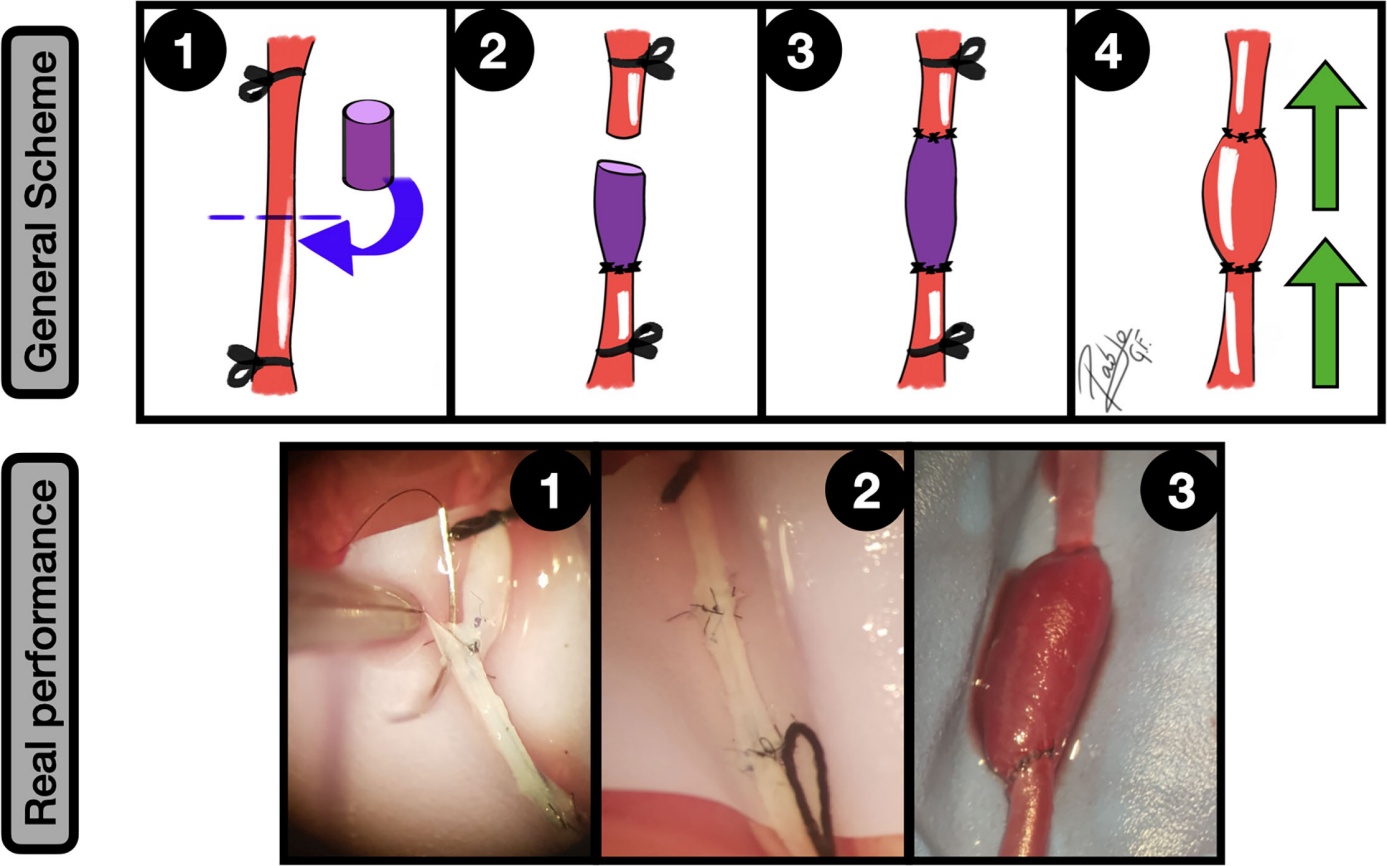
Fusiform aneurysms. General scheme: **1:** Unilateral CCA exposure and proximal and distal ligation. A straight arteriotomy in the middle of the artery is done and a free venous graft from a jugular vein is harvested; **2:** One of the free lumens is sutured to the caudal CCA segment in an end-to-end fashion; **3:** A second end-to-end anastomosis is made between the graft and the proximal CCA, acting as an interpositional venous graft; **4:** The clamps are released and the flow inflates the venous segment of the construction, obtaining a fusiform aneurysm. The green arrows indicate the blood flow direction. Real performance: 1: Suturing between CCA proximal segment and the free venous graft; **2:** The second end-to-side anastomosis is completed, observing the interpositional venous graft; **3:** After releasing the clamps the blood flow enlarges the venous segment of the construction, simulating a fusiform aneurysm.

The non-saccular aneurysms (fusiform, dolichoectatic, and dissecting) are, by far, less common than saccular among human IAs (38), and the available data revealed internal elastic lamina fragmentation, thinning of the tunica media and tunica intima hyperplasia as was shown in their saccular counterparts (4), although their exact pathogenesis remains unclear. The potentially tortuous arterial pathway makes this translational model more suitable to train endovascular skills than microsurgical clipping. Most of the fusiform models of IAs are described on bifurcation aneurysm (4, 36, 38, 39, 54), adding some modifications to the graft harvesting to enlarge the saccular dilatation longitudinally. The previously described tenets are valid for the creation of such exercise, in which often a venous graft is preferred over an arterial (4, 52). However, there is a chance to create by microsurgery a pure fusiform aneurysm on a straight continuous segment of a vessel (6, 55), which may resemble those found, for instance, in human intracranial vertebral arteries. The fusiform aneurysm is obtained immediately or in a short period, in part due to the graft properties, which allows obviating a long time to develop a defective elastic lamina and muscular layers observed in human fusiform IAs (56). While the description is made for CCA as follows, this model can be performed in any of the three vascular compartments of the rat (Figure 10):

1. Jugular vein patch harvesting, CCA exposure, clamping, and straight arteriotomy
2. Interpositional venous graft placement on CCA and distal suture in an end-to-end fashion
3. A double end-to-end anastomosis is completed by a second proximal end-to-end anastomosis
4. After the clamping is released, the venous pouch enlarges progressively whilst the blood flows through the lumen, resembling a fusiform aneurysm.

Fukui et al. (56) described this model on the rat CCA, by using an interpositional femoral vein graft, achieving a dramatic fusiform enlargement in 75% and a formation of a giant aneurysm in 53% of the grafts.

Other described modifications of this simple fusiform model include an additional branch (6) emerging from the aneurysm that can contribute to improve the outflow and potentially avoid thrombosis.

#### Other Complex Aneurysms

The aforementioned types of extracranial models for IAs in rats do not include all the possibilities in terms of anastomoses combination, but they are by far the most common types reported in the literature. Other simulated aneurysms are classified into this group under the name “other complex aneurysms” by some authors (18), attending to unusual shapes resembling several types of human IA, such as dolichoectatic, giant-sized, fusiform, bisaccular (57), or confluence artery aneurysm. The obtained creations are mostly oriented to endovascular therapy training and new device testing rather than microsurgery training, and for this reason, these publications are preferentially found in journals focused on interventional neuroradiology (47, 57). Due to the number of involved vessels and resulting size, these models are unpopular to be practiced in rodents, being more frequently described in bigger animals, such as rabbits or pigs (18).

They are not well-characterized and there are many variations between models, being the design many times ultra-specific (47) with regards to the bifurcation angle, size and complex vessels flow circuitry that allows the trainee to practice sequential temporal clipping to decrease, for instance, the inflow into the aneurysm or to assess the enlargement of the dome when the outflow is clamped.

The giant aneurysm model provides a good example of this class, where enlargement of a huge formation that sometimes needs a week to be appreciated, and that may also be performed at one stage.

Some of the simulations recreate the vessels circuitry found in the human vertebrobasilar system, where the two vertebral arteries bring a blood inflow into a common basilar trunk that later divides into another two vessels: the posterior cerebral arteries. Thanks to these complex models, some specific portions and flow conditions of human intracranial vessels can be simulated, offering a simplified model to understand and assess the clipping practice.

**Figure 11:**
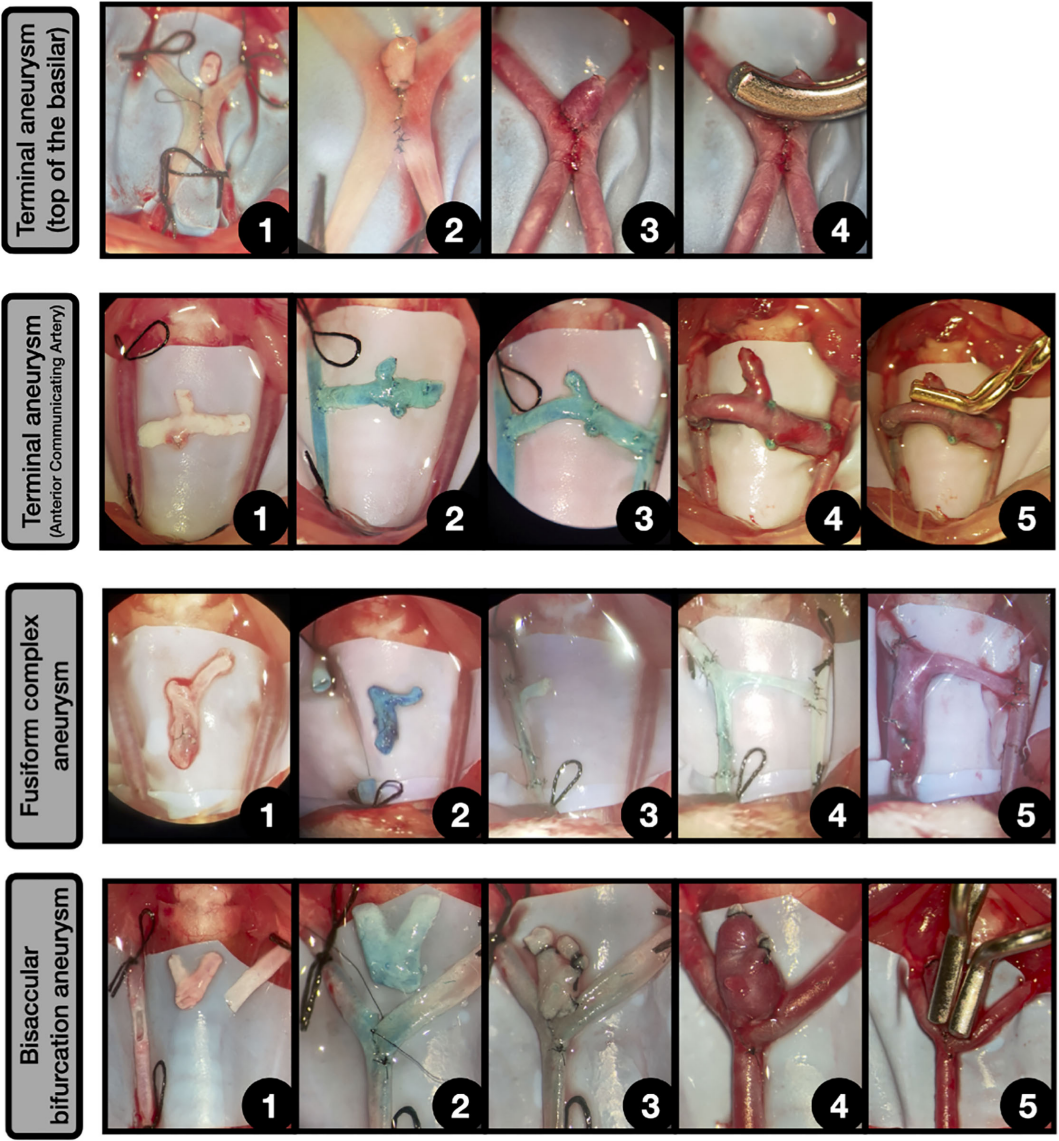
Other complex aneurysms. Terminal aneurysm (Top of the basilar) [4]: **1:** Side-to-side anastomosis between both CCAs, leaving a hole in the top. Above lies a free venous graft; **2:** The graft is sutured to the hole and the free edge is occluded; **3:** After declamping the aneurysm enlarged on the top of the anastomosis, simulating a terminal aneurysm placed on the top of the construction; **4:** The aneurysm is clipped. Terminal aneurysm (Anterior communicating artery) [4]: **1:** Both CCAs are exposed and ligated and a free graft from jugular vein bifurcation is obtained; **2:** The graft is anastomosed in an end-to-side fashion to the right CCA; **3:** Another end-to-side anastomosis between the graft and the contralateral CCA is performed, obtaining a connection between both CCAs. The remaining above free lumen is occluded by suture; **4:** After releasing the ligatures a terminal aneurysm that arises from the venous graft is obtained, simulating the circuitry of an anterior communicating artery; **5:** The aneurysm is clipped. Fusiform complex aneurysm [4]: **1:** Both CCAs are exposed and ligated and a free graft from jugular vein bifurcation is obtained; **2:** A straight arteriotomy is made in right CCA and the free venous graft is dyed in blue; **3:** The graft is anastomosed in an end-to-end fashion to both CCAs free lumens, leaving a branch oriented to the contralateral CCA; **4:** The branch is anastomosed to the contralateral CCA in an end-to-side fashion; **5:** After the declamping, the venous segment enlarges, obtaining a complex fusiform aneurysm. Bisaccular bifurcation aneurysm [77]: **1:** Longitudinal arteriotomy in the parent vessel and oblique fish-mouth arteriotomy in the second vessel for an end-to-side anastomosis. A bifurcation venous graft, obtained from the jugular vein is placed on the surgical field; **2:** Incomplete end-to-side anastomosis between both vessels, leaving a hole on the top; **3:** The venous graft is anastomosed to the top of the construction and the two free lumens are occluded; **4:** After the declamping, the venous graft enlarges, obtaining a complex bifurcation bisaccular aneurysm; **5:** The aneurysm is clipped by using two aneurysm clips.

Here is provided a brief summary of some other complex aneurysms which can be crafted on rodents' vessels model (Figure 11).

## Discussion

### Historic Perspective of the Microsurgical Training in Neurosurgery

Surgical training was long based on experimental models, being the animals' involvement in medicine a constant throughout history. The refinement of the microscopes and the advances in the vascular neurosurgery discipline have followed parallel tracks, being impossible to understand both mentioned areas without the presence of each other. A century ago, in 1922, the Swedish otologist Gunnar Holmgren introduced the first binocular surgical microscope in operating theater (58), setting the beginning of a new era for surgery that will take more than 30 years to arrive at Neurosurgery, when, in 1957 the first reported microsurgery attempt in the field took place, by Theodore Kurze, who removed a neurilemoma of the seventh nerve in a child. At the same time, in Vermont, in 1958, R. Donaghy (59) established the world's first microsurgery research and training laboratory for neurosurgeons, after the series production of the Zeiss OpMi 1 started, in the early 50s. His growing interest in the field pushes him and his colleague Jacobson, to report their translational accomplishments in neurovascular surgery, describing in detail the instrumentation and technique for microsurgical reconstruction of small intracranial arteries in a case series (60), which is considered the first human reported microvascular experience in neurosurgery. Prior to this date, in 1939, German and Taffel had documented the first experimental encephalomyosynangiosis in dogs and primates, followed by Kredel in 1942, who did the first attempt in humans (61). However, these revascularization procedures are indirect techniques that were performed without the use of the microscope, essentially because its use was not yet standardized. By 1965, Dr. Donaghy's laboratory had attained global renown, receiving many physicians from all over the world who wanted to learn the microsurgery standards and take part in his annual 2-weeks course (62). One of the most remarkable surgeons among these trainees was G. Yaçargil, whose restless dedication to microneurosurgery will bring huge advances and contributions to the field. He highlighted the need for reproducible animal models to develop microvascular techniques (63), by transferring and adapting the original one described by Alexis Carrel (64) (Nobel Prize 1912), and by introducing specific novel instruments for microsurgery, including the aneurysm clips and appliers or the Malis bipolar coagulation, which was a turning point in his career, as he recognized further. In this sense, his first steps were made on the rabbit femoral artery and dog carotid artery, where he achieved a 66% anastomosis patency rate (61) during his training period at R. Donaghy's laboratory until they purchased a microscope for his department. Nowadays, the best outcomes are achieved by an accepted patency rate of over 95% for extracranial-intracranial bypass procedures, bringing to focus the indispensable resource of microvascular training (61).

In the 1960s and 1970s, thanks to the efforts of many pioneers in the field, the refinement of the techniques was possible, and many new techniques were born, allowing to improve the odds against several craniospinal diseases that were previously considered untreatable. This was the case of Woringen and Kunlin, who performed the first extracranial-intracranial bypass in a real patient by 1963 (65).

The weight of the historical evidence indicates that the major advances in the field of vascular neurosurgery came from the technical improvement and application of microsurgery principles, being further applied to other sub-disciplines such as oncological surgery or spine surgery. Furthermore, it is well-known the association between microvascular training in living models and the development of the most subtle surgeries among the greatest neurosurgeons, who encourage the young generations to follow their steps by routinely practicing on these models (1).

### Learning in Microsurgery

Microsurgery is known as a high-demanding discipline where routinely training is indispensable to achieve the best results in the real world, a fact that in modern practice becomes more important than ever due to extremely high expectations toward surgeons (15). Many of the best neurosurgeons of the second half of twentieth century have hypothesized about the importance of cadaveric and animal model training. Contrary to what some people may think, microsurgery training is not about the anastomosis final result, but the essence of microsurgery, and therefore, of bypass surgery too, are the micromovements (1). These maneuvers are mainly based on the hand-eye coordination (or better said: “hand-brain”) needed to carry on the finest vascular suturing.

At present, a varied range of training publications takes the quality and number of movements into account, moving the exercise results into the background. This fact is reflected, for instance, in the increasing need to quantify microsurgery training by using scales that attempt to measure these subjective parameters. Despite of the fact that there is still a lack of standardization, some of the main scales compile the following issues (5, 11): self-confidence, theoretical knowledge [Global Rating Scale (GRS) and Northwestern Objective Microanastomosis Assessment Tool (NOMAT)], subjective self-assessment or by a third party [Objective Structured Assessment of Technical Skills (OSATS), University of Western Ontario microsurgical skills acquisition/assessment (UWOMSA), GRS, and NOMAT] (66), objective motion control (The Stanford Microsurgery and Resident Training (SMaRT)) (67), analysis of the final result of the anastomosis (UWOMSA, GRS, and NOMAT), time to complete anastomosis (UWOMSA, GRS) and transferability.

The ideal scale must probably include all these issues (5), however, the high heterogeneity among the available scales in terms of measuring tools, makes it difficult to compare prospective results.

It is also critical a suitable training model selection, adapted to the level and skills of the trainee, who must begin on non-living dry models during the practice of the most basic steps (11) until enough expertise to try on more realistic and living models had been gained. Likewise, the complexity of exercises to be performed in microsurgery varies with the practitioner skills, being recommended a sequential learning program, which means performing and solidifying the knowledge of the most basic steps and anastomoses at the beginning. Then the trainee can advance (5) to other training stages where it is imperative to dominate different types of anastomoses, vessels, and exercises that culminate in complex combinations such as aneurysms performances.

A high-level surgeon does not obtain only a good result, but he looks for good stable results. There are some studies (68) that emphasize the importance of ongoing training for mastering the technique, and compile all these questions, setting over 50 the number of anastomoses as a threshold to master the microsurgery technique.

**Figure 12:**
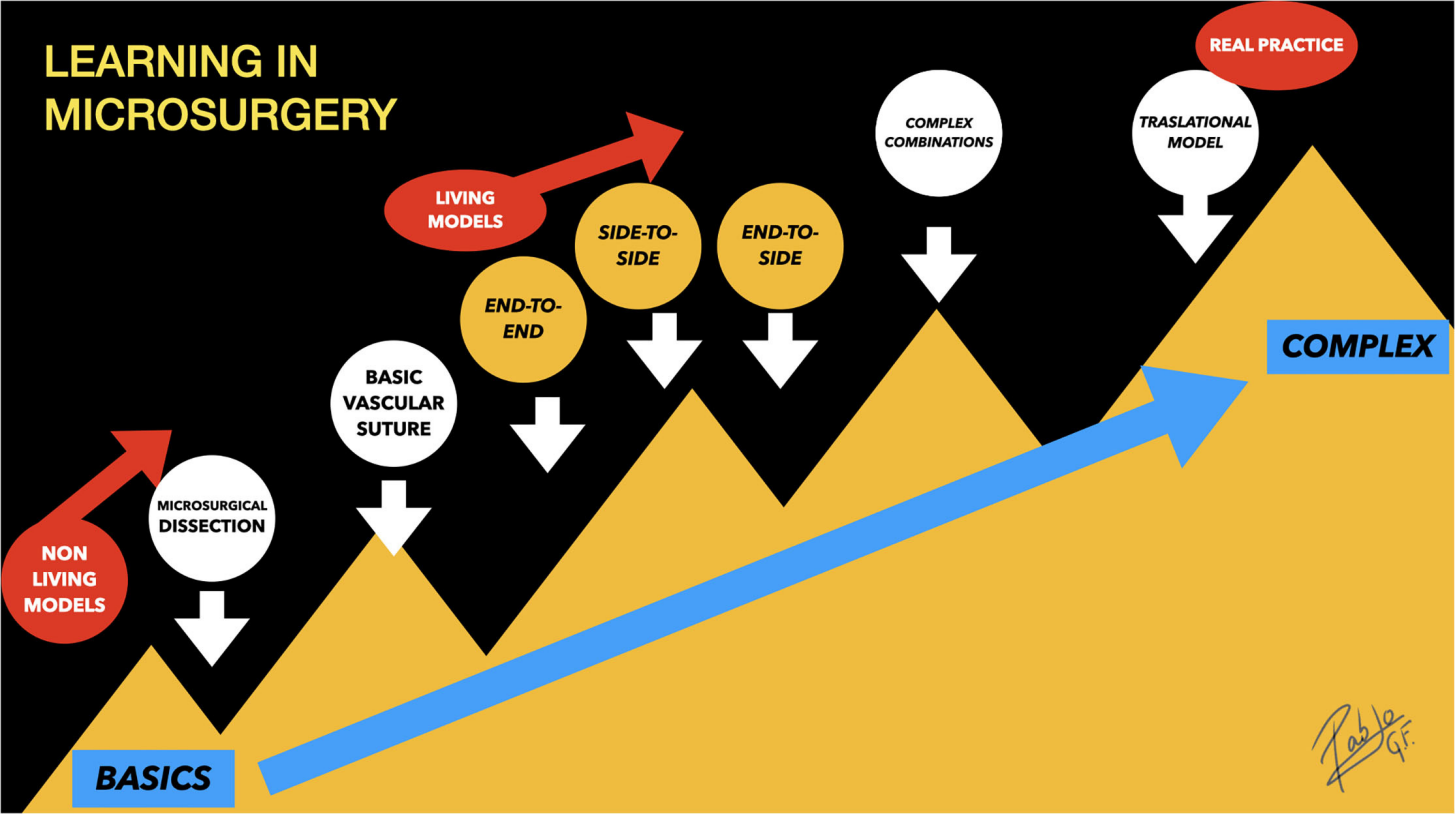
Learning in microsurgery: Stepwise training route for learning in microsurgery. The end-to-side and side-to-side anastomoses are a little harder to be performed than end-to-end, which is considered the simplest anastomosis. The aneurysm performances are located on the top of the complexity, under the name “Complex Combinations,” that refers to their underlying microarchitecture that includes portions of the classic three anastomoses.

The proposed stepwise training route (Figure 12) for learning in microsurgery is based on the current published recommendations, highlighting the value of the three classical anastomoses technical execution handicap (end-to-end, end-to-side, and side-to-side), which are the basis of experimental microsurgery (1, 5, 11, 68), so as to make possible the further development of the aneurysm creation models, where these techniques are mandatory to be mastered.

### A Realistic Model With Limitations

Although the described aneurysm models are technically more demanding, they enable vascular clipping practice, inflow and outflow evaluation, growing patterns assessment, and, in some cases, the flow can be regulated.

The rodent vessels model for IAs is more predictable with regard to size and volume (13, 46) in comparison to induction models, being the neck, angle, and orientation potentially fashioned. The final average volumes are similar to those found in human IAs (35.19 ± 5.64 mm^3^) (13), especially to those found in the posterior communicating artery or the postero-inferior cerebellar artery, being useful these trained skills, to our knowledge, during a tumor resection involving posterior circulation vessels, like in medulloblastoma surgery. However, some human aneurysms arising from anterior circulation can achieve a large size that cannot be obtained easily by the mentioned training exercise.

They are not good models for rupture, presenting most of them the increases in size by means of maturation rather than ongoing degradation of the aneurysm wall and true growth that finally results in aneurysm rupture (69). Despite these differences, they are quite good models to train on suturing of an injured aneurysm neck and microsurgical reparation of vessels (69).

In rodents, the surrounding tissue differs from the human arachnoid layers, typically seen during cranial approaches, being the aneurysms located on the three vascular compartments of the rodent, between fatty tissue and abdominal organs. Furthermore, neither induction nor creation models can accurately replicate the real pathophysiology observed in IAs in terms of, for example, atheromatosis (20), timing, or rupture trends.

Unfortunately, none of the available models offer all the ideal features of a good aneurysm model, which should include the following: tissue responses, stability without spontaneous thrombosis when untreated, perianeurysmal environment, physical dimensions, minimal surgical, and endovascular morbidity; similarity to human aneurysm shear stresses, hemodynamic forces, and physical dimensions (70).

It is known that the three classical anastomoses are the pillars of microsurgery training (5, 68), that have been extensively utilized in real patients during by-pass surgery, however, some differences in several aspects from aneurysm creation models are noticed:

- The absence of a direct application for real cases since the creation of an artificial intracranial aneurysm in a human has no sense.
- Sometimes, the artificial connection created between the vessels causes an arteriovenous fistula, a condition of unclear utility in human intracranial vessels.
- A longer ischemia time in comparison to a simple anastomosis exercise.

The last point is of considerable interest, understood as the time required for the aneurysm creation, which is related to the time without blood flow. Some authors reported shorter creation times for the simplest models (sidewall, bifurcation stump, and natural bifurcation), which can take around 30–60 min (18). Some publications (13) have even reported 15–20 min of ischemia for the natural bifurcation aneurysm model. However, the full invested time, considering all the steps, such as the graft harvesting and preparation of the parent vessels, is frequently longer than the time for a simple anastomosis, being often necessary 3 h of surgery to performed artificial bifurcation aneurysms (13, 18).

There remains considerable controversy regarding human arterial occlusion time, as it is described in one of the most powerful conducted studies about cerebral Occlusion Surgery Study) (71). This study reports a mean duration of MCA occlusion, among the 78 patients who did not experience a stroke, within 2 days after by-pass surgery of 45.4 ± 24.2 min, ranging from 15 to 123 min. Surprisingly, this study revealed no statistical significance (*p* = 0.182) in terms of MCA occlusion time compared to the stroke cohort. Despite the results, neurosurgeons worldwide still recommend the shortest possible ischemia time during bypass procedures. We believe that this time is essential too, and we encourage the reader to practice on the described models to shorten their by-pass/aneurysm creation time as much as possible.

The rodent model is not ideal for endovascular training (simpler aneurysm creations, shorter vessels caliber, and smaller size), since more realistic aneurysms can be performed in swine or rabbit model, which both offer bigger vessels.

### Comparison Between Aneurysm Models

The described aneurysm models are suitable for several research and training-related uses, however, there are many differences between induction and creation models, besides its mechanism of production, that becomes a matter of ongoing interest for the present review. Most of the mentioned induction models in rodents consist of an addition of different risk factors, that are also observed in humans, and that lead to IA incidence (20).

It is known that the costs of rodents are cheaper than the swine, canine o rabbit models, where, additionally, general anesthesia and the presence of a veterinary must be required (18). Furthermore, the costs of the induction models are normally higher, due to the requirement for several drugs administration, the use of knock-out cohorts, or the time needed for an acceptable IA incidence rate, which in some cases can last up to 6 (20) or 13 months (37). Via the creation model, the aneurysm is obtained in a moment. Moreover, in terms of aneurysm shape, the most common IAs induced in rodents are non-rounded or bulges consisting of small outpouchings arising from the animal intracranial vessels (20), normally located in the anterior circulation, that are not suitable to be surgically manipulated due to their size. Whilst in induction models neither the incidence rate nor the aneurysm shape can be predicted, creation models offer several fashionable characteristics such as size, location, type, longitudinal axis length, neck width, aneurysm projection, bifurcation angle, dome-to-neck ratio, and other dome peculiarities (13). All these designable properties make them ideal for training since more similar aneurysms in comparison to human IAs can be achieved, despite being placed on extracranial rodent vessels (18). The hemodynamic circuitry can be also designed, becoming important, for instance, for the bifurcation aneurysms understanding, where the blood flow direction determinates whether a bifurcation whether a confluence aneurysm (46) is created, a subtle distinction that provides a vertebrobasilar junction or an MCA bifurcation aneurysm prototype.

Only two of the described non-pure creation models provide a training opportunity for microsurgical purposes: the Hashimoto induction model (29) and the fusiform aneurysm induction model on CCA (38). The first suggests an end-to-side anastomosis between both CCAs (to secondarily induce IAs), which must be meticulously performed in order to ensure the animal can survive the procedure for several weeks (29–31). The second one implies a gentle dissection of the CCA and its microsurgical isolation before a local high dose administration of elastase (38).

Sidewall aneurysms are the quickest to be performed and they normally imply fewer vessels manipulation. For instance, the terminal branch occlusion model just requires microsurgical dissection and ligation of the main vessel, avoiding a microvascular anastomosis. The complex models are not very popular in rodents, and their angioarchitecture is very variable with poor reproducibility, being more suitable for bigger animals, where the caliber of the vessels allows for novel endovascular devices testing (18).

Probably the most complete models to train on vascular skills are the ones that include several types of vessels (both venous and arterial), where a modified anastomosis is demanded.

More than one creation model exercise can be tried on the same animal, leading to the reduction of the number of employed animals, agreeing with the 3Rs principles proposed by Russell and Burch (16).

### New Horizons in Vascular Training

Several milestones have been reached since the introduction of the microscope in the surgical routine one century ago (58), evolving from the macroscopic era into the microneurosurgical era. Despite these accomplishments, since the beginning of the twenty-first century, there is a sustained declining trend of the number of microsurgical clipping and by-pass procedures, motivated by some big trials results such as ISAT (International subarachnoid aneurysm trial) (72) which included a huge cohort of randomized patients suffering aneurysmatic SAH, suggesting lower short-term mortality in the endovascular treatment arm (23.5 vs. 30.9%; 95% CI 3.6–11.2; *p* = 0.0001) when compared to surgical clipping arm. However, a *post hoc* analysis issued eighteen years after (73) invalidated these results, showing no differences between both arms. Finally, in 2012, BRAT (The Barrow Ruptured Aneurysm Trial) (3) was published, highlighting the importance of high-quality surgical clippings when the endovascular treatment has failed and enhancing the need of both surgical and endovascular provisioned centers to achieve the best outcomes. These investigations encouraged new generations to be ready for neurovascular surgery procedures, however, the current fewer vascular surgical cases, which many times are more complex, makes essential the training on different models.

The classic living models continue to be the gold standard for training among senior vascular surgeons, however, the scientific society claims for the refinement of the technique and the avoidance of living animals for training purposes, so new devices are increasingly emerging as alternatives to supply these “old-school” models. Some authors have tried to develop virtual surgery simulation (4), a promising method that presents the main inconvenience of the lack of haptic feedback, typically seen during the *in vivo* models surgical clipping. This handicap has motivated the development of several prototypes of haptic devices for medical use that calculate the required force, returning a calculated proportional response to the user in real-time, as is the case of *Bimanual Haptic Simulator for Medical Training, PalpSim*, or *ImmersiveTouch* (12). This field of research is now more active than ever, promising major advances in the coming decades that will further reduce the human-machine interface so that virtual simulations are more realistic. An example is the recent *NeuroVR*^*TM*^ *Platform*, which integrates a 3D rendering system with binocular output, emulating a surgical microscope, with a bimanual haptic rendering (12).

*MicroSure* (74) is one of the robots in which the practice of microvascular anastomoses has been evaluated in a live rodent model, by successfully performing end-to-end anastomoses in the aorta and femoral arteries, but still obtaining a suboptimal aesthetic aspect of construction. The use of robots in microsurgical practice requires a certain degree of prior training, in which the typical haptic feedback of conventional microsurgery is dropped. At present, concerning neurosurgery (74), the brachial plexus repair and the sympathetic chain repair to treat Horner's syndrome have been documented, but not any vascular intracranial procedure.

Silicone aneurysm models offer an excellent alternative to animals in both endovascular and clipping training; however, it does not fully replicate the natural arterial biology (36). 3D-printed technologies allow the presurgical accurate recreation of the patient-specific vessels, which can be further processed to form a hollow, silicone-walled artificial vasculature for training and serve as an assessment tool for vascular cases, including even, some of them, a device that induce a simulated blood flow (75, 76).

## Conclusion

Among twenty-first century neurosurgeons, the lowest complications rate and the perfect refinement of the vascular technique are pointed (15), so a hard training program is mandatory to be completed before starting vascular procedures in real patients.

A stepwise training program with regards to the trainee skills and exercises complexity must be taken into account before starting experimental surgery training involving living models.

The presented aneurysm models suggest new training exercises, different from the classic anastomoses, that can be reproduced following the explained steps and that allowed further realistic clipping due to many similarities to those IAs found in humans.

The present review summarizes the present state-of-the-art microsurgical techniques for the development of both intracranial and extracranial aneurysms in rodents, which are still valid, representing the best training for the vascular by-pass skills reinforcement as a translational model useful for neurovascular surgeons, that can also help to improve the underlying mechanism of the pathophysiology of human IAs. By training on these models, several microsurgical skills are improved, allowing safer practice for neurosurgeons in all stages of their career.

## Author Contributions

FC and AI provided substantial contributions to the conception and analysis of the work by their experience. MS-A provided a critical analysis of some portions of the review. MG helped during redaction and provided a critical analysis of the review. All authors contributed to the article and approved the submitted version.

## Funding

This article submission was supported by the grant PI17/00361 by instituto Carlos III of Spanish Ministry of Health and MULTITARGET & VIEW by Community of Madrid (FEDER).

## Conflict of Interest

The authors declare that the research was conducted in the absence of any commercial or financial relationships that could be construed as a potential conflictof interest.

## Publisher's Note

All claims expressed in this article are solely those of the authors and do not necessarily represent those of their affiliated organizations, or those of the publisher, the editors and the reviewers. Any product that may be evaluated in this article, or claim that may be made by its manufacturer, is not guaranteed or endorsed by the publisher.

## Abbreviations

IA: Intracranial aneurysm
SAH: Subarachnoid hemorrhage
CCA: Common carotid artery
MCA: Middle cerebral artery
AIB: Aorto-iliac bifurcation.
